# Efficacy of Vein of Marshall Ethanol Infusion Added to Left Atrial Anatomical Ablation for Treatment of Persistent Atrial Fibrillation in Patients with Hypertrophic Cardiomyopathy

**DOI:** 10.31083/j.rcm2410302

**Published:** 2023-10-23

**Authors:** Tao Luo, Tao Liu, Bo Cui, Xi Li, Jinlin Zhang, Gang Wu

**Affiliations:** ^1^Department of Cardiology, Renmin Hospital of Wuhan University, 430060 Wuhan, Hubei, China; ^2^Cardiovascular Research Institute, Wuhan University, 430060 Wuhan, Hubei, China; ^3^Hubei Key Laboratory of Cardiology, 430060 Wuhan, Hubei, China; ^4^Department of Cardiology, Wuhan Asian Heart Hospital, 430060 Wuhan, Hubei, China

**Keywords:** catheter ablation, vein of marshall, hypertrophic cardiomyopathy, persistent atrial fibrillation, pulmonary vein isolation

## Abstract

**Background:**

Radiofrequency catheter ablation (RFCA) has been shown to have low efficacy for the treatment of persistent atrial fibrillation (AF) in patients with hypertrophic cardiomyopathy (HCM). We conducted this study to evaluate the benefit of adjunctive vein of Marshall (VOM) ethanol infusion during RFCA for persistent AF (PsAF) in patients with non-obstructive HCM.

**Methods:**

This multicenter retrospective observational study included 102 consecutive non-obstructive HCM patients with PsAF who underwent RFCA plus VOM ethanol infusion (VOM-EI) (RFCA + VOM, n = 56) or RFCA alone (RFCA, n = 46) for the first time. The efficacy endpoint was survival without AF or atrial tachycardia (AT) after the blanking period.

**Results:**

We completed the VOM-EI in 92.9% (52/56) patients. The left pulmonary vein antrum ablation time (RFCA + VOM: 19.9 ± 6.1 min vs. RFCA: 27.2 ± 9.3 min), mitral isthmus (MI) ablation time (RFCA + VOM: 16.9 ± 3.7 min vs. RFCA: 28.4 ± 7.8 min), and rate of coronary sinus (CS) vein ablation (RFCA + VOM: 57.69% vs. RFCA: 80.43%) were lower but the acute success rate of MI block (RFCA + VOM: 98.1% vs. RFCA: 84.8%) were higher in the RFCA + VOM group than those in the RFCA group (all *p* < 0.05). After twelve months follow-up, 84.6% of patients (44/52) survived without AF/AT in the RFCA + VOM group, compared to 65.2% of patients (30/46) in the RFCA group (*p* = 0.03; odds ratio = 2.93, 95% CI: 1.18–7.79).

**Conclusions:**

VOM-EI combined with RFCA decreased the recurrence rate of AF/AT at 12 months in HCM patients with PsAF. VOM-EI simplified the ablation of the left pulmonary vein antrum and MI and increased the success rate of MI bidirectional block.

## 1. Introduction

Atrial fibrillation (AF) is commonly observed as a result of left atrial dilation and remodeling in patients with hypertrophic cardiomyopathy (HCM), with a prevalence of >20% [1, 2]. AF increases the risk of stroke and worsens the symptoms of heart failure, resulting in increased major adverse clinical events in HCM patients [1, 2, 3]. Emerging evidence suggests that early rhythm control can provide more favorable treatment outcomes in patients with AF [4]. Unfortunately, the available pharmacological options for permanently maintaining sinus rhythm (SR) are compromised due to potential side effects and suboptimal efficacy in patients with AF [5]. AF ablation is a safe and optimal alternative to antiarrhythmic drug (AAD) therapy for SR maintenance and symptom improvement [6]. Pulmonary vein isolation (PVI) remains the cornerstone of catheter ablation for AF. Additional linear ablation beyond PVI is often the recommended AF ablation strategy for persistent AF (PsAF). However, in patients with HCM and PsAF, PVI alone or PVI plus linear ablation has been shown to have relatively low efficacy for the restoration and maintenance of SR [7, 8, 9]. The vein of Marshall (VOM) is innervated, triggers AF, and can be ablated by ethanol perfusion. The VENUS trial (The Vein of Marshall Ethanol for Unablated Persistent AF (VENUS) trial) revealed that catheter ablation plus VOM ethanol infusion (VOM-EI) improved the ablation outcomes in PsAF [10]. However, the effects of adding VOM-EI to radiofrequency catheter ablation (RFCA) in patients with HCM and PsAF have not yet been investigated. Therefore, we studied the efficacy of adjunctive VOM-EI during left atrial anatomical ablation for PsAF in non-obstructive HCM patients.

## 2. Materials and Methods

### 2.1 Study Design

This was a multicenter, retrospective, observational study that included consecutive adult non-obstructive HCM patients with PsAF between January 2018 and December 2021 who underwent VOM-EI combined with RFCA or RFCA alone for the first time. We defined HCM as a left ventricular myocardium thickness of ≥15 mm based on a two-dimensional echocardiogram [11]. PsAF was defined as an AF episode lasting beyond seven days and an episode terminated by cardioversion after seven days [6].

### 2.2 Periprocedural Management

Before the procedure, an electrocardiogram, computed tomography angiogram of the heart, Holter monitoring, and transthoracic echocardiography were performed. Left atrial thrombus was excluded using transesophageal echocardiography. Anticoagulation therapy was interrupted on the day of the procedure and continued 4–6 hours after the procedure, with pericardial effusion excluded.

### 2.3 General Anesthesia

All patients underwent general anesthesia before the VOM-EI and RFCA procedures. Anesthesia was induced by intravenous administration of etomidate, remifentanil, and benzene sulfonyl atracurium. After endotracheal intubation, anesthesia was maintained using intravenous dexmedetomidine, remifentanil, and sevoflurane inhalation. Sedation level and state of consciousness were monitored using the bispectral index, which was maintained at 40–60 during the procedure.

### 2.4 VOM Ethanol Infusion Procedure 

**Fig. 1.**
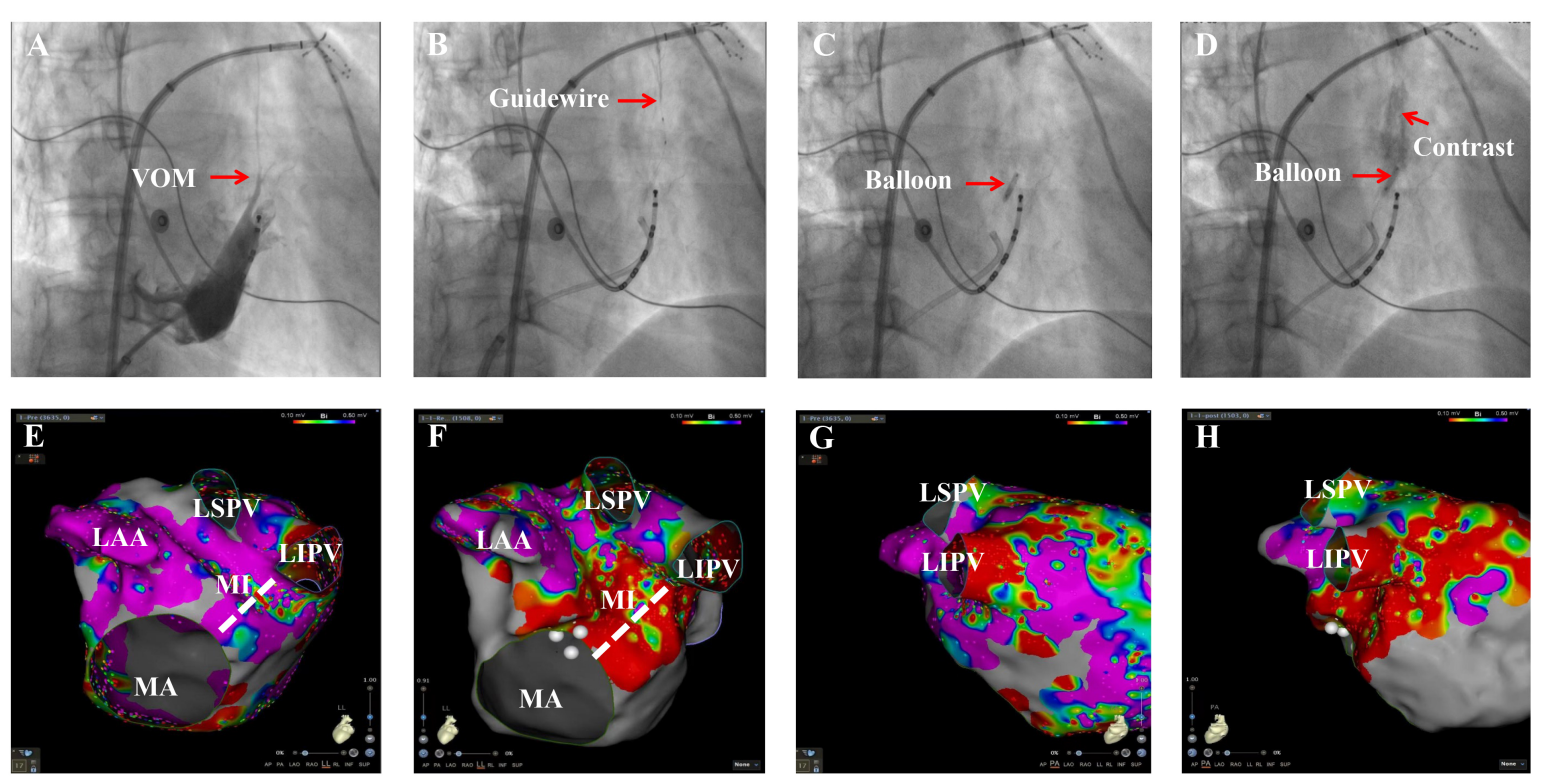
**The procedural steps of the vein of Marshall (VOM) ethanol infusion (VOM-EI).** (A) Angiography of the VOM in 30° right anterior oblique (RAO) views. (B) Guidewire in the distal end of the VOM. (C) The proximal end of the VOM sealed by the dilational balloon. (D) Ethanol-infused tissue was stained. (E) Voltage mapping of the left pulmonary veins, left pulmonary vein-LAA ridge region, and the posterior before VOM-EI in left lateral (LL) views. (F) Voltage mapping showed low-voltage regions in the posterior MI and left pulmonary vein-LAA ridge region, after VOM-EI. (G) Voltage mapping of LIPV before VOM-EI in posteroanterior (PA) views. (H) Voltage mapping in LIPV showed low-voltage regions after VOM-EI. LAA, left atrial appendage; LSPV, left superior pulmonary vein; LIPV, left inferior pulmonary vein; MI, mitral isthmus; MA, mitral annular. The red arrow marks the VOM in Fig. 1A, the guidewire in Fig. 1B, the balloon in Fig. 1C, and the contrast agent and the balloon in Fig. 1D, respectively. The white dashed line marks the MI in Fig. 1E,F.

The VOM-EI procedure was routinely administered before the RFCA procedure in all patients. Intravenous heparin administration maintained an activated clotting time of 300–350 seconds. After transseptal punctures, three-dimensional anatomical reconstruction and the high-density voltage mapping of the left atrium (LA) were performed using a Pentaray Nav eco high-density mapping catheter (Biosense Webster, Diamond Bar, CA, USA) under the guidance of the CARTO3 electroanatomical mapping system (Biosense Webster, Diamond Bar, CA, USA). Baseline left atrial bipolar voltage mapping was performed in the AF rhythm in all patients, and any low voltage was noted. In patients in the VOM + RFCA group, voltage mapping was repeated after VOM-EI and limited to the ethanol-infused VOM region. The color scale ranged from 0.1 to 0.5 mV. A voltage below 0.1 mV was defined as scared myocardium, and a voltage above 0.5 mV was defined as normal myocardium. The region where the bipolar voltage was <0.5 mV was defined as the low-voltage area (LVA), and its size was obtained by manual measurement. VOM-EI was performed stepwise according to a previously published procedure [12]. Briefly, a Webster Fixed Curve Catheter (Biosense Webster, Ciudad Juarez, Chihuahua, Mexico) was advanced into the coronary sinus (CS) through the left subclavian vein. A long Swartz sheath (Abbott Medical, Nathan Lane North Plymouth, MN, USA) was advanced through the right femoral vein into the CS. A 6F JR 3.5 guiding catheter (Cordis US Corp., Ciudad Juarez, Chihuahua, Mexico) was advanced through the long Swartz sheath to inject contrast and place a guide wire. The VOM was visualized by CS vein angiography in 30° right anterior oblique (RAO) views (Fig. 1A). After the angiographic visualization of the VOM, a guidewire preloaded with a MUSTANG^TM^ OVER-THE-WIRE dilated balloon catheter (Boston Scientific Corporation, Maple Grove, MN, USA) was advanced through the 6F JR 3.5 guiding catheter. The guidewire was gently advanced to the distal end of the VOM to provide sufficient support for balloon movement (Fig. 1B). VOM angiography was performed before ethanol infusion to ensure that the proximal end of the VOM was sealed with a dilational balloon (Fig. 1C). A total of 12 mL of anhydrous alcohol was then injected using a 3-mL syringe divided over 4 applications via the inflated balloon catheter. After repeated VOM ethanol infusions, the area of the ethanol-infused tissue was stained using fluoroscopy (Fig. 1D). The balloon was deflated, and the entire system was removed after the final ethanol infusion. Voltage mapping was performed on the anatomical location of the VOM, including the left pulmonary veins, left pulmonary vein-left atrial appendage (LAA) ridge region, and posterior mitral isthmus (MI), before (Fig. 1E,G) and after VOM-EI (Fig. 1F,H).

### 2.5 Radiofrequency Catheter Ablation Procedure

The RFCA procedure was performed using a thermocool SMARTTOUCH SF (ST SF) uni-directional navigation catheter (Biosense Webster, Ciudad Juarez, Chihuahua, Mexico) in a power-controlled mode (power 45 W; saline irrigation 15 mL/min; temperature 43 °C). Radiofrequency applications were displayed automatically using the VisiTag module (Biosense Webster, Diamond Bar, CA, USA) with predefined location stability (maximum distance change of 5 mm within 3 seconds) and minimum force (5 g for at least 70% of the time). RFCA primarily involves PVI and additional linear ablation. Briefly, the first step was to perform circumferential pulmonary vein ablation to achieve PVI according to the ‘CLOSE’ protocol [13]. As previously described [14], the entrance and exit block in SR confirmed PVI. Additional linear ablation and bidirectional block, including the LA roof and MI lines were performed in the second step of the RFCA procedure. PVI and additional linear ablation were guided by the ablation index values (ridge 450, inferior 400, superior 400, posterior 400, roof line 400, MI line 500, and cavotricuspid isthmus (CTI) line 450). Differential pacing on both sides of the ablation line confirmed the MI bidirectional block according to the criteria [15]. CS vein ablation (power 30 W; saline irrigation 15 mL/min) was performed if the MI was not blocked. Linear MI and CS vein ablations were performed under fluoroscopy to improve the success rate and reduce the complication rate of the MI block. For patients with CTI-dependent atrial flutter, CTI linear ablation and bidirectional block were performed. A bidirectional CTI block was confirmed by differential pacing from the inferior lateral wall of the right atrium and the proximal CS. Activation mapping-guided ablation was performed if atrial tachycardia (AT) was generated during ablation. After achieving PVI and completing linear ablation, direct current cardioversion (bi-phase 150–200 J) was performed to restore the SR if the patient was still in the AF rhythm. AT was induced by burst pacing in the proximal CS with a decreasing pacing cycle length. If AT was generated, activation mapping-guided ablation was performed. The desired endpoint of the procedure was the termination of AF.

### 2.6 Post-Procedural Management

All patients were administered an oral proton pump inhibitor for 4 weeks after the procedure to prevent esophageal injury. AADs, including amiodarone and beta-blockers, were administered three months after the procedure, after which amiodarone was discontinued. The long-term administration of beta-blockers is recommended to improve HCM symptoms.

### 2.7 Follow-Up

Through outpatient visits, follow-up was scheduled at 1, 3, 6, and 12 months after the procedure. Additional outpatient visits and further testing were immediately performed at any time when recurrent arrhythmia symptoms occurred. Visits included a physical examination, electrocardiogram, Holter or 14-day single-lead electrocardiogram monitoring, and assessment of clinical status and current antiarrhythmic medication. The first three months post-procedure were defined as the blanking period. The relapse of AF/AT during the blanking period has also been documented. Recurrence was defined as AF/AT occurring >30 s after the blanking period. The absence of AF/AT relapse throughout the follow-up period was considered as freedom from AF/AT survival. The efficacy endpoint was survival without AF/AT after blanking. The following complications were documented for safety: pericardial tamponade, pericardial effusion not requiring drainage, vascular complications, thromboembolism, phrenic nerve palsy, esophageal fistula, and procedure-related death.

### 2.8 Statistical Analysis

All study data were analyzed using IBM SPSS 26.0 (IBM Corp., Chicago, IL, USA). Continuous normally distributed variables were expressed as mean ± standard deviations (SD) and analyzed by Student’s *t*-test. Categorical variables were presented as percentages and were analyzed using the Fisher’s exact test. Kaplan-Meier curves were constructed to illustrate freedom from atrial arrhythmias, and the log-rank *p* test was used to evaluate the differences between the two groups. Statistical significance was set at *p* < 0.05 (two-tailed).

## 3. Results

### 3.1 Characteristics of Patients

A total of 102 HCM patients were included in two electrophysiology centers between January 2018 and December 2021. Fifty-six patients were in the RFCA + VOM group, and 46 patients were in the RFCA group. In the RFCA + VOM group, VOM-EI was successfully performed in 92.9% (52/56) of patients. Four patients were excluded from the RFCA + VOM group due to failed VOM ethanol infusions. The baseline characteristics of the patients with HCM in each group are shown in Table 1. The two groups had no significant differences in demographics, comorbidities, or clinical data.

**Table 1.** **The baseline characteristics of the patients with HCM**.

		RFCA + VOM	RFCA	*p* value
		(n = 52)	(n = 46)	
Age, years		60.9 ± 7.7	61.5 ± 7.2	0.69
Male sex, n (%)		37 (71.2)	31 (67.4)	0.83
Body mass index		26.0 ± 2.7	25.4 ± 2.8	0.28
CHA2DS2-VASc score		2.3 ± 1.6	2.14 ± 1.2	0.58
Hypertension, n (%)		28 (53.8)	25 (54.3)	0.83
Diabetes mellitus, n (%)		12 (23.1)	9 (19.6)	0.81
Prior stroke or transitory ischemic attack, n (%)		8 (15.4)	6 (13.0)	0.78
Coronary artery disease, n (%)		15 (28.9)	11 (23.9)	0.65
Time from first AF diagnosis				
	<6 months, n (%)	15 (28.8)	12 (26.1)	0.82
	6 months to 2 years, n (%)	19 (36.5)	16 (34.8)	>0.99
	>2 years, n (%)	18 (34.6)	18 (39.1)	0.68
NYHA class				
	I, n (%)	41 (78.9)	35 (76.1)	0.81
	II, n (%)	9 (17.3)	8 (17.4)	>0.99
	III, n (%)	2 (3.8)	3 (6.5)	0.66
AAD therapy				
	Beta-blocker	41 (78.8)	39 (84.8)	0.60
	Amiodarone	21 (40.4)	22 (47.8)	0.54
	Glomerular filtration rate, mL/min	82.8 ± 15.3	81.6 ± 11.0	0.66
	NT-proBNP, pg/mL	1809.9 ± 918.9	1783.8 ± 865.5	0.89
	LA diameter, mm	46.0 ± 4.3	46.3 ± 4.1	0.73
	Septal left ventricular thickness, mm	16.2 ± 1.4	15.9 ± 1.2	0.26
	Posterior wall left ventricular thickness, mm	11.9 ± 1.1	11.7 ± 1.3	0.41
	left ventricular ejection fraction, %	55.4 ± 2.3	54.9 ± 2.5	0.31
Mitral regurgitation				
	No, n (%)	20 (38.5)	19 (41.3)	0.84
	Mild, n (%)	19 (36.5)	15 (32.6)	0.83
	Moderate, n (%)	10 (19.2)	10 (21.8)	0.81
	Severe, n (%)	3 (5.8)	2 (4.3)	>0.99

Data are shown as mean ± SD or absolute numbers and percentages (n%). RFCA, radiofrequency catheter ablation; VOM, vein of Marshall; NYHA, New York Heart Association; NT-proBNP, N-terminal pro-B-type natriuretic peptide; LA, left atrium; HCM, hypertrophic cardiomyopathy; AF, atrial fibrillation; AAD, antiarrhythmic drug.

### 3.2 Procedural Data

The procedural data are shown in Table 2. Total fluoroscopy and procedural times were similar between the two groups. Successful PVI and LA roof blocks were achieved in all patients in each group. Compared with the RFCA group, lower left PVI time (19.9 ± 6.1 min vs. 27.2 ± 9.3 min, *p* < 0.0001) and MI linear ablation time (16.9 ± 3.7 min vs. 28.4 ± 7.8 min, *p* < 0.0001), a higher acute success rate of MI block (98.1% [51/52] vs. 84.8% [39/46], *p* = 0.02), and a lower rate of CS vein ablation (57.69% [30/52] vs. 80.43% [37/46], *p* = 0.02) were observed in the RFCA + VOM group. No significant differences in the rates of CTI ablation, conversion to ATs, or conversion to SR were found between two the groups. The two groups had no significant differences in the total size of the baseline left atrial LVA (4.6 ± 5.1 vs. 3.6 ± 4.8 ${\mathrm{cm}}^{2}$, *p* = 0.73). In the RFCA + VOM group, the total size of the left atrial LVA after VOM-EI was significantly higher than before (19.1 + 6.1 vs. 4.6 + 5.1 ${\mathrm{cm}}^{2}$, *p* = 0.0004). In the RFCA + VOM group, VOM-EI directly resulted in left inferior PVI in 12 of 52 patients and achieved MI block in 5 of 52 patients. The total procedural time of the VOM-EI was 21.2 ± 10.3 min and required 6.8 ± 4.2 min of fluoroscopy.

**Table 2.** **Procedural data in each group**.

	RFCA + VOM	RFCA	*p* value
	(n = 52)	(n = 46)	
Total procedural time, min	150.8 ± 23.3	151.9 ± 28.8	0.84
Total fluoroscopy time, min	21.7 ± 5.6	22.8 ± 7.3	0.40
Successful PVI, n (%)	52 (100)	46 (100)	>0.99
Left PVI time, min	19.9 ± 6.1	27.2 ± 9.3	<0.0001
LA roof block obtained, n (%)	52 (100)	46 (100)	>0.99
MI ablation time, min	16.9 ± 3.7	28.4 ± 7.8	<0.0001
MI block obtained, n (%)	51 (98.1)	39 (84.8)	0.02
CS vein ablation, n (%)	30 (57.69)	37 (80.43)	0.02
CTI ablation, n (%)	5 (9.6)	6 (13.0)	0.75
Conversion to ATs, n (%)	9 (17.3)	7 (15.2)	0.78
Conversion to SR, n (%)	8 (15.4)	6 (13.0)	0.78

Data are shown as mean ± SD or absolute numbers and percentages (n%). RFCA, radiofrequency catheter ablation; VOM, vein of Marshall; PVI, pulmonary vein isolation; LA, left atrium; MI, mitral isthmus; CS, coronary sinus; CTI, cavotricuspid isthmus; AT, atrial tachycardia; SR, sinus rhythm.

### 3.3 Adverse Events

The incidence of complications in patients with HCM was relatively low, regardless of whether they received RFCA plus VOM-EI or RFCA alone. Overall, the two groups had similar adverse events (Table 3).

**Table 3.** **Adverse events in each group**.

	RFCA + VOM	RFCA	*p* value
	(n = 52)	(n = 46)	
Overall adverse events, n (%)	1(1.9)	1(2.2)	>0.99
Pericardial tamponade, n (%)	0	0	
Pericardial effusion not requiring drainage, n (%)	0	1(2.2)	0.47
Vascular complications, n (%)	1(1.9)	0	>0.99
Thromboembolism, n (%)	0	0	
Phrenic nerve palsy, n (%)	0	0	
Atrioesophageal fistula, n (%)	0	0	
Procedure-related death, n (%)	0	0	

Data are shown as absolute number and percentage (n%). RFCA, radiofrequency catheter ablation; VOM, vein of Marshall.

### 3.4 Clinical Outcomes

**Fig. 2.**
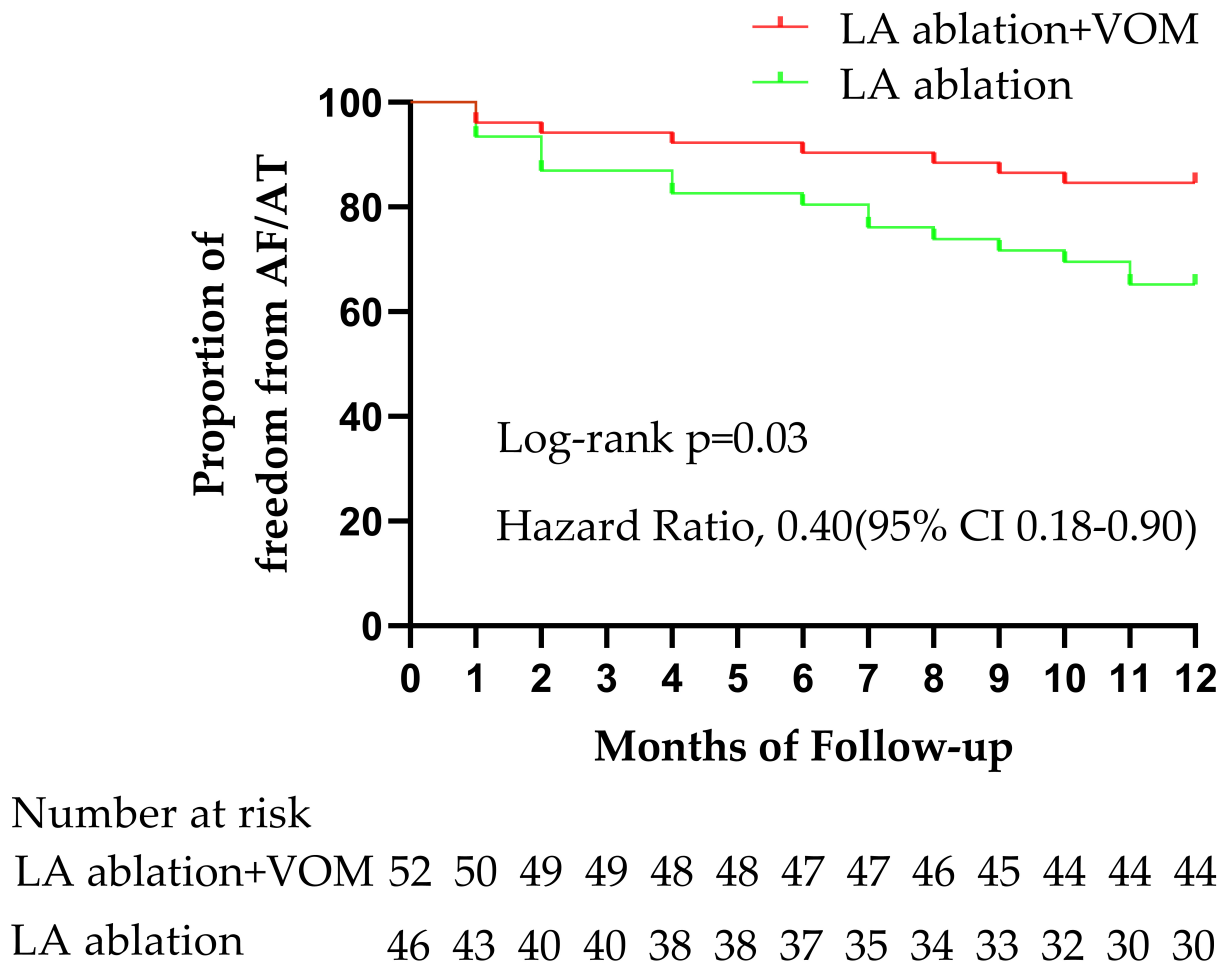
**AF/AT -free survival curve after the different ablation procedures.** The survival curve in the RFCA + VOM and the RFCA groups was traced by red or green, respectively. RFCA, radiofrequency catheter ablation; VOM, vein of Marshall; AF, atrial fibrillation; AT, atrial tachycardia; LA, left atrium.

Twelve months post-procedure, the survival without AF/AT after the blanking period was 84.6% (44/52) in the RFCA + VOM group and 65.2% (30/46) in the RFCA group (odds ratio = 2.93; 95% CI: 1.18–7.79; *p* = 0.03) (Fig. 2, Table 4). In the RFCA + VOM group, eight of 52 (15.4%) patients had AF/AT recurrences, including five patients with AF and three patients with AT. In the RFCA group, 16 of 46 (34.8%) patients had AF/AT recurrences, including six patients with AF and ten patients with AT. The rate of recurrence with AT was lower in the RFCA + VOM group than in the RFCA group (RFCA + VOM: 21.7% [10/46] vs. RFCA: 5.8% [3/52], *p* = 0.03) (Table 4). Among patients with recurrence, five patients in the RFCA + VOM group underwent redo ablation, including one of five (20.0%) patients with AT recurrence due to a gap in the MI line. In contrast, eight patients from the RFCA alone group underwent redo ablation, including five of eight (62.5%) patients with AT recurrence due to a gap in the MI line. In the patients who underwent repeat ablation, the restoration rate of MI conduction was higher in the RFCA alone group than in the RFCA + VOM group; however, this increase was not statistically significant (*p* = 0.27).

**Table 4.** **Main clinical outcomes**.

	RFCA + VOM	RFCA	Odds ratio	*p* value
	(n = 52)	(n = 46)	(95% CI)	
Freedom from AF/AT following the blanking period, n (%)	44 (84.6)	30 (65.2)	2.93 (1.18–7.79)	0.03
Recurrence, n (%)	8 (15.4)	16 (34.8)	0.34 (0.13–0.85)	0.03
Recurrence as AF, n (%)	5 (9.6)	6 (10.9)	0.71 (0.22–2.72)	0.75
Recurrence as AT, n (%)	3 (5.8)	10 (21.7)	0.22 (0.06–0.87)	0.03

Data are shown as absolute number and percentage (n%). RFCA, radiofrequency catheter ablation; VOM, vein of Marshall; AF, atrial fibrillation; AT, atrial tachycardia.

## 4. Discussion

### 4.1 Main Findings

The data from our multicenter study represent one of the largest cohorts of patients with HCM who received RFCA and VOM-EI for the treatment of PsAF. Our research demonstrated the following: (1) compared with RFCA alone, RFCA combined with VOM-EI increased AF/AT-free survival at the 12-month follow-up in HCM patients with PsAF; (2) VOM-EI was associated with facilitation of left PVI and MI linear ablation; and (3) VOM-EI is safe and feasible for PsAF treatment in HCM patients.

### 4.2 Strategy of Atrial Fibrillation Ablation in Patients with Hypertrophic Cardiomyopathy

AF can cause loss of atrial systolic function, diastolic dysfunction, and shortened filling time, leading to decreased tolerance of patients with HCM to AF and increased mortality in HCM patients [3]. Therefore, when AF occurs, SR and subsequent long-term rhythm control are highly desirable in patients with HCM. In patients with HCM, AAD treatment does not consistently maintain SR owing to its potentially dangerous side effects and relatively low efficacy [1, 5]. Previous studies have confirmed that AF ablation is an important treatment for patients with HCM; however, the risk of recurrence is twice that in the general population [16]. A recent multicenter observational study showed unfavorable outcomes in 137 HCM patients with HCM who received AF RFCA for AF. In this study, after an average 3-year follow-up, almost all HCM patients with PsAFs experienced recurrence of atrial arrhythmia, despite one-third of the patients having undergone treatment with AAD [8]. Additionally, in a recently published meta-analysis, only 46.1% of patients with HCM and PsAF after a single AF ablation were free from atrial arrhythmias during the 12 months of follow-up [9]. In our study, 12 months after a single AF ablation, 65.2% of the HCM patients with PsAF had no AF/AT recurrence. In our study, the success rate of AF ablation was higher than that reported in previous studies. The VOM contains innervation, myocardial junctions, and arrhythmogenic foci, and is associated with the pathogenesis of AF [17]. The myocardium adjacent to the VOM and its innervation can be ablated using retrograde balloon intubation and ethanol infusion [17]. The VOM-EI may improve SR maintenance by enhancing atrial denervation, achieving a more reliable MI bidirectional block, or eliminating triggers [17, 18]. A recent clinical trial showed that RFCA combined with VOM-EI improved outcomes in patients with PsAF [10]. However, the clinical data on VOM-EI during ablation for AF in patients with HCM remains limited. Therefore, this retrospective study included a large cohort of patients with HCM who received RFCA combined with VOM-EI (n = 52) or RFCA alone (n = 46) for the treatment of PsAF. Our data demonstrated the benefits of adding VOM-EI to conventional AF ablation. The overall ablation success rate exceeded our expectations, with 84.6% of HCM patients having no recurrence of AF/AT after 12 months of follow-up in the RFCA + VOM group (44 of 52), compared with 65.2% (30 of 46) in the RFCA group.

Theoretically, additional VOM-EI could increase the procedural and fluoroscopy times compared with RFCA alone. In this study, the total procedural time of the VOM-EI was 21.2 ± 10.3 min and required 6.8 ± 4.2 min of fluoroscopy. We found that VOM-EI resulted in a large number of LVAs in areas consistent with VOM distribution, including the left inferior pulmonary vein, left pulmonary vein-LAA ridge region, and posterior MI. To achieve a higher success rate for the MI block, we performed the “point-by-point” MI linear ablation and CS vein ablation under fluoroscopy. VOM-EI directly resulted in left inferior PVI in 12 of 52 patients and achieved MI block in 5 of 52 patients from the RFCA + VOM group. VOM-EI also significantly reduced the rate of CS vein ablation by 22.74% in the RFCA + VOM group compared to that in the RFCA alone group. The ablation time for the left pulmonary vein and MI and the fluoroscopy time for MI ablation were markedly shorter in the RFCA + VOM group than in the RFCA alone group. Therefore, the fact that the total time of fluoroscopy exposure and procedural time were similar between the two groups might be attributed to VOM-EI, which simplifies the ablation of the left pulmonary vein and MI.

PVI and linear ablation failed to improve the success rate of PsAF, primarily because of an incomplete block of the MI. When performing ablation for MI, achieving a bidirectional block via an endocardial approach is often challenging, and CS vein ablation is generally required. The VOM is an anatomical structure located in the epicardial aspect of the posterior MI, between the left inferior pulmonary vein (LIPV) and CS, providing an epicardial approach for ablation of the MI [17]. VOM-EI has been proven to be associated with a shorter ablation time for MI and a higher success rate for MI block in a previous study [19]. In our study, VOM-EI was associated with a significantly shorter left PVI time, a shorter MI ablation time, a significantly higher success rate of acute MI block, and significantly lower CS vein ablation rate. The VENUS trial also demonstrated the facilitation of left PVI and MI ablation, which benefits from VOM-EI [10].

In this study, compared with RFCA alone, VOM-EI added to RFCA significantly increased the acute success rate of the MI block by 13.3%. However, the acute success rate of the MI block was 84.8% in the RFCA alone group. It seems that the efficacy of VOM-EI was not dependent on achieving MI block. Thirteen patients with relapse underwent repeat ablation in our study. Among these patients, one of five (20.0%) in the RFCA + VOM group and five of eight (62.5%) in the RFCA alone group had AT recurrence due to a gap in the MI line. The restoration rate of MI conduction was higher in the RFCA alone group than in the RFCA + VOM group. However, the difference between the two groups was not statistically significant, possibly owing to the small sample size. We speculated that VOM-EI could increase the long-term success rate of the MI block, thereby decreasing the AF recurrence in our study. The long-term impact of VOM-EI combined with RFCA on the MI block needs to be further studied with larger sample sizes.

The incidence of AT after ablation for AF is relatively high. AT occurs in 31% of patients after PVI, as reported by Deisenhofer* et al*. [20]. Notably, 58% of the patients in this study had structural heart disease [20]. In contrast, in a recently published study, more than one-third of patients with HCM developed AT after AF ablation [7]. MI-dependent atrial flutter is common after AF ablation, with rates as high as 33–60%. The VOM is located within the myocardium of the MI, critical for maintaining The VOM is located within the myocardium of the MI, critical for maintaining MI-dependent atrial flutter [17]. The Marshall ligament is a degenerate epicardial fold made up of the VOM and myocardium tissue called the Marshall bundle (MB). In patients with left atrioventricular tachycardia after AF ablation, up to 30.2% of ATs are MB-mediated. Treatment of this type of arrhythmia often requires ablation of the MB-LA or CS-MB junction or infusion of ethanol into the VOM [21]. In our study, recurrence of AT during 12 months of follow-up was reached in 5.8% of patients (3/52) in the RFCA + VOM group and 21.7% of patients (10/46) in the RFCA group (*p* = 0.03), with an odds ratio of 0.22 (95% CI: 0.06–0.87). AT recurrence following AF ablation is common in patients with HCM. Therefore, VOM-EI may be essential for reducing the incidence of AT after AF ablation.

Since it was first performed in May 2008 in a patient undergoing PsAF ablation, VOM-EI has been proven to be a valid, feasible, and safe therapy for AF ablation [17]. Although additional time was required to complete the VOM-EI, the total procedural and fluoroscopy times did not increase significantly in our study. No complications directly related to the VOM-EI procedure were observed.

### 4.3 Limitations

Our study had several limitations. First, this was a retrospective, observational study. Secondly, we may have missed some asymptomatic recurrences because we did not perform continuous electrocardiogram monitoring. Third, a follow-up of 12 months was relatively short. Future studies should evaluate long-term effects of VOM-EIs. Fourth, our data only represents a subset of the HCM population, as we did not include patients with obstructive HCM in this study. Finally, in our study, isoproterenol infusion was not performed after PVI alone or VOM ablation, which confirmed effective PVI and evaluated the appearance of non-pulmonary vein triggers.

## 5. Conclusions

Among HCM patients with PsAF, adjunctive VOM-EI decreased the recurrence rate of AF/AT at 12 months compared to RFCA alone. VOM-EI facilitated the ablation of the left pulmonary vein antrum and MI and improved the success rate of the MI block. VOM-EI may be essential for reducing the incidence of AT after catheter ablation for AF in patients with HCM. Adjunctive VOM-EI during catheter ablation for AF may be a safe and feasible strategy for patients with HCM.

## Data Availability

The datasets used and/or analyzed during the current study are available from the corresponding author on reasonable request.

## Abbreviations

AF, atrial fibrillation; HCM, hypertrophic cardiomyopathy; SR, sinus rhythm; AAD, antiarrhythmic drug; PVI, pulmonary vein isolation; PsAF, persistent atrial fibrillation; VOM, vein of Marshall; VOM ethanol infusion, VOM-EI; RFCA, radiofrequency catheter ablation; LA, left atrium; CS, coronary sinus; LAA, left atrial appendage; MI, mitral isthmus; CTI, cavotricuspid isthmus; AT, atrial tachycardia; MB, Marshall bundle; NYHA, New York Heart Association; NT-proBNP, N-terminal pro-B-type natriuretic peptide.
